# Impacts of drought stress on soluble carbohydrates and respiratory enzymes in fruit body of *Auricularia auricula*

**DOI:** 10.1080/13102818.2014.984522

**Published:** 2014-11-26

**Authors:** Huai-liang Ma, Xiu-hong Xu, Xiao-yu Zhao, Hua-jing Liu, Huan Chen

**Affiliations:** ^a^ Department of Applied Microbiology, College of Resource and Environment, Northeast Agricultural University, Harbin, Heilongjiang, P.R. China; ^b^ Department of Biotechnology, College of Life Science and Technology, Mudanjiang Normal University, Mudanjiang, Heilongjiang, P.R. China

**Keywords:** *Auricularia auricula*, drought stress, soluble carbohydrates, respiratory enzyme, fruit bodies

## Abstract

In order to study the survival mechanisms to drought stress for fruit body of *Auricularia auricula*, soluble carbohydrates and respiratory enzymes were investigated. Fruit bodies were exposed to sunlight and were naturally dehydrated. Samples were taken at different levels of water loss (0%, 10%, 30%, 50% and 70%) to measure the content of soluble sugars and polysaccharides. The activities of phosphoglucose isomerase (PGI), combined glucose-6-phosphate dehydrogenase (G-6-PDH) and 6-phosphogluconate dehydrogenase (6-PGDH), and malate dehydrogenase (MDH), were also determined. The results showed that with the increase in water loss, soluble sugars and MDH activity declined, whereas the activities of G-6-PDH and 6-PGDH increased. Soluble polysaccharides content and PGI activity decreased with water loss up to 30% and increased afterwards. These results suggested that the pentose phosphate pathway (PPP), as demonstrated by activities of G-6-PDH and 6-PGDH, could be one of the mechanisms for survival during drought stress in the fruit body of *A. auricula*. Moreover, soluble polysaccharides may play a part in protecting the fruit body in further drought stress.

## Introduction

*Auricularia auricula*, known as the wood ear or jelly ear, is a species of edible macrofungus cultivated worldwide, especially in China. To achieve higher yields of *A. auricula*, cultivar breeding [1] and cultivation techniques [2,3] have been studied extensively. In addition, environmental conditions for cultivation, such as temperature, water, air humidity, light and O_2_ are essential to production of *A. auricula.*[4] Among environmental conditions, water and air humidity play important roles in cultivating *A. auricula*. Fruit bodies grow well at 90%–95% relative air humidity and 60%–65% water content in substrates.[4] However, wild or cultivated *A. auricula* is frequently subjected to drought stress (relative air humidity below 80% and water content below 60% in substrates), which is considered the most important factor that limits the fruit body growth and yield.

Drought stress reduces the water potential of organisms and produces excessive levels of reactive oxygen species (ROS), such as O_2_·^−^, H_2_O_2_, ^1^O_2_, OH·, which are extremely toxic and trigger membrane lipid peroxidation and rapid destruction of cellular constituents, resulting in oxidative stress and causing cell injury or death.[5–7] This is particularly true for plants. When the intensity and duration of the drought stress increase, plants will wither and die. Surprisingly, the fruit bodies of *A. auricula* are different from plants. They dry easily in drought stress, but restore their growth, once water and air humidity are available. What mechanisms are involved in the survival during drought stress is still poorly understood. Compared to plants, much fewer studies are available on the physiology and survival mechanisms of the fruit body of *A. auricula* during drought stress. The knowledge about the physiology and survival mechanisms is crucial for the cultivation of *A. auricula* and is of great significance to understanding the surviving strategies in macrofungi.

Respiration is a central part of the metabolism and is of great physiological importance to aerobic organisms, because it provides energy, reducing power, e.g. reduced nicotinamide adenine dinucleotide (NADH), reduced nicotinamide adenine dinucleotide phosphate (NADPH) and reduced flavin adenine dinucleotide (FADH_2_), and intermediates for other metabolic pathways. There are three major pathways of respiration: the Embden–Meyerhof pathway (EMP) or the glycolytic pathway, the tricarboxylic acid cycle (TCAC) and the pentose phosphate pathway (PPP). Currently, the contribution of respiration to drought tolerance is not well understood and more researches are still required.

In the present study, we determined the changes in soluble carbohydrates and respiratory enzymes during dehydration of the fruit bodies of *A. auricula*, with the aim to investigate the role of respiration in the survival mechanism to drought stress.

## Materials and methods

### Cultivation and treatments

*A. auricula* strain Techan 2 was obtained from the Institute of Forest By-Product and Speciality (Mudanjiang, China). The substrates for cultivating *A. auricula* contained sawdust (80%), wheat bran (10%), rice bran (6%), corn meal (2%), gypsum (l%), lime (1%) and their water content was 65%. The substrates were filled in polyethylene bags (17 cm × 33 cm). After being autoclaved at 121 °C for 1.5 h, the bags were inoculated with 0.5% grain spawn of *A. auricula* and cultivated at 25 °C in total darkness. One hundred holes (5 mm in diameter) were punched on the surface of the bags when they were filled with mycelia and then the bags were placed in open fields and watered thrice per day. When the fruit bodies grew to about 2.0 cm in diameter, they were picked, exposed to sunlight and naturally dehydrated. Samples were taken when water loss was 0% (the control) or reached 10%, 30%, 50% and 70%. The water loss [%] was calculated by the following formula: water loss = [(initial weight − final weight)/initial weight] × 100.

### Soluble carbohydrates

Soluble sugars were extracted according to Saito et al. [8] with some modifications. A fruit body (0.5–1.0 g) was ground under ice-cold conditions in 20 mL of 80% (v/v) ethanol. The homogenate was extracted three times in water bath at 80 °C for 20 min with shaking. After centrifugation at 8500 × *g* for 15 min, the supernatants were combined and used for the determination of soluble sugars.

To extract the soluble polysaccharides, the residue from the ethanol extraction was subsequently suspended in 20 mL of distilled water and extracted three times with boiling water for 1 h.[9] The extracts were collected after centrifugation at 8500 × *g* for 15 min and were used to assay the soluble polysaccharides.

Soluble sugars were determined according to Saladin et al. [10]. Briefly, 0.2 mL of ethanol extract were mixed with 1 mL of anthrone–sulphuric acid reagent (0.1% anthrone (w/v) and 0.1% thiourea (w/v) in 12.5 mol L^−1^ sulphuric acid) and incubated at 100 ^o^C for 10 min. After cooling, the absorbance was measured at 625 nm. Soluble polysaccharides were quantified by the method of Dubois et al.[11] One millilitre of polysaccharides solution (diluted appropriately) was mixed with 1 mL of 5% phenol (w/v) and 5 mL of concentrated sulphuric acid. The mixture was shaken and placed in a water bath at 25 °C for 30 min and then the absorbance was determined at 490 nm. We used glucose as a standard during the measurement of both soluble sugars and soluble polysaccharides. The results were expressed as milligrams of glucose equivalents per gram (dry weight, DW) of fruit body.

### Respiratory enzymes activities

A fruit body (0.5–1.0 g) was ground in 20 mL of 0.1 mol L^−1^ Tris-HCl buffer (pH 7.4), using a chilled mortar and pestle. The homogenate was centrifuged at 10,000 × *g* for 15 min at 4 °C and the supernatant was used for enzymatic analysis. All enzymes activities were determined at 25 °C.

Phosphoglucose isomerase (PGI) activity was assayed by the Thomas’ procedure [12] with minor modifications for measuring the production of fructose-6-phosphate. The reaction solution contained 0.1 mL of the enzyme extract and 0.9 mL of 5 mmol L^−1^ glucose-6-phosphate (prepared in 0.1 mol L^−1^ Tris-HCl buffer). The solution was kept at 25 °C for 10 min and the reaction was stopped by addition of 3.0 mL of concentrated HCl and 1.0 mL of 1% resorcinol (w/v, prepared with 95% ethanol). The mixture was incubated at 80 °C for 10 min and cooled rapidly to room temperature. The absorbance of the mixture was measured at 530 nm. The fructose-6-phosphate content was calculated by comparison with a standard curve of fructose.[13] One unit of activity was defined as the amount of enzyme producing 1 μmol fructose-6-phosphate per minute.

Combined activities of glucose-6-phosphate dehydrogenase (G-6-PDH) and 6-phosphogluconate dehydrogenase (6-PGDH) were measured using Brown and Wray's method,[14] with some modifications. The assay medium contained the following components: 0.1 mol L^−1^ Tris-HCl buffer (pH 7.4); 5 mmol L^−1^ glucose-6-phosphate; 5 mmol L^−1^ NADP^+^ and 5 mmol L^−1^ MgCl_2_. Enzyme extract (0.1 mL) was mixed with 0.9 mL of the assay medium. Then, the absorbance of the mixture was recorded at 340 nm every 30 s. NADPH content was calculated by using the millimolar extinction coefficient of 6.22 L mmol^−1^ cm^−1^. One unit of activity was defined as the amount of enzyme that caused the formation of 1 μmol NADPH per minute.

Malate dehydrogenase (MDH) activity was assayed based on Thomas’ method,[12] with minor modifications. The assay solution included the following components: 0.1 mmol L^−1^ Tris-HCl buffer (pH 7.4), 5 mmol L^−1^ NADH and 10 mmol L^−1^ oxaloacetate. The absorbance was recorded at 340 nm every 30 s, after the addition of 0.1 mL of enzyme extract in 0.9 mL of assay solution. NADH content was quantified by using the millimolar extinction coefficient of 6.22 L mmol^−1^ cm^−1^. All enzyme activities were expressed as units per milligram protein. Protein was estimated by the method of Coomassie brilliant blue G250 described by Bradford,[15] using bovine serum albumin as a standard.

### Statistical analysis

Each measurement was performed with five replications and the results were expressed as mean ± standard deviations (SD). If the data satisfied normality and equal variances, they were analysed by one-way analysis of variance (ANOVA), followed by Duncan's multiple range test (DMRT) for multiple comparisons. In addition, analysis of variance was conducted by using the Kruskal–Wallis H test and multiple comparisons were assessed by *t*-test. A value of *P* < 0.05 was considered to be significant.

## Results and discussion

### Soluble carbohydrates

**Figure 1.**
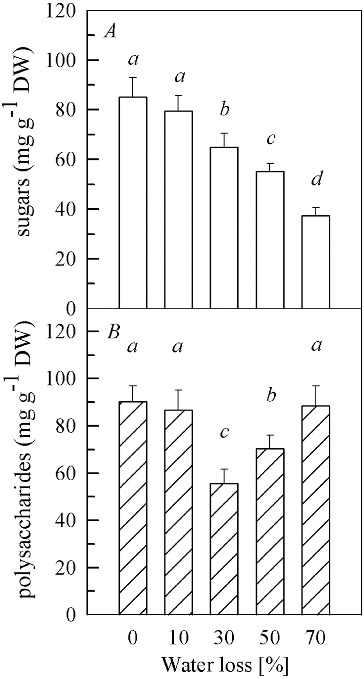
Contents of soluble sugars (A) and soluble polysaccharides (B) in fruit bodies of *Auricularia auricula* under water loss. Mean values from five replications. Error bars represent ± SD. Different lowercase letters indicate significant difference (*P* < 0.05).

There was no difference in the content of soluble sugars (Figure 1(A)) between the samples with 10% water loss and the control (0% water loss). When the water loss increased from 30% to 50% and 70%, the levels of soluble sugars significantly decreased (*P* < 0.01) and were 76%, 65% and 44% of those in the control, respectively. The content of the soluble polysaccharides (Figure 1(B)) also remained unchanged at 10% water loss, compared to the control. However, it fell to its minimum (62% of the control) (*P* < 0.01) at 30% water loss and then significantly increased to 78% of the control (*P* < 0.01) at 50% of water loss. When the water loss reached 70%, the content of soluble polysaccharides increased (*P* < 0.01) to the level of the control.

The increase of compatible solutes (also termed osmoprotectants), especially soluble sugars, is well documented in plants and plays an important part in the stabilization of the cell membranes and proteins during drought stress.[16–20] However, in our study, the concentration of soluble sugars showed a different trend. Thus, soluble sugars were not used as an osmoprotectant to protect fruit bodies, but it seemed that they served as substrates for respiratory metabolism under drought stress. However, the increased levels of soluble polysaccharides at 30%–70% water loss may participate in osmoregulation and be helpful to resist further drought stress.

### Respiratory enzyme activities

**Figure 2.**
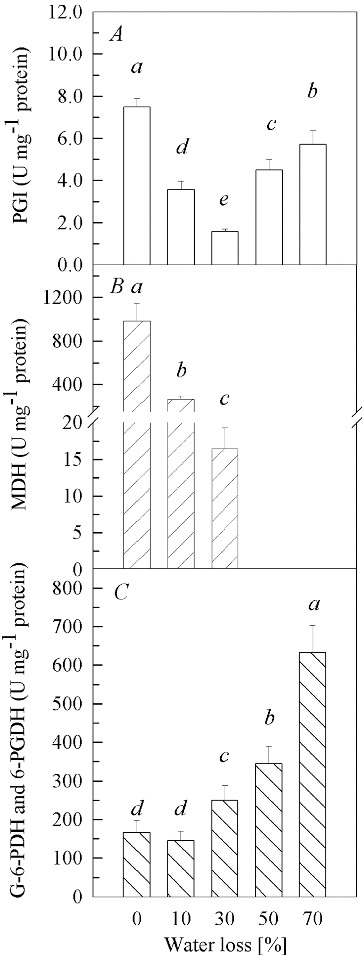
Activities of PGI (A), MDH (B) and G-6-PDH and 6-PGDH (**C**) in fruit bodies of *Auricularia auricula* under water loss. Mean values from five replications. Error bars represent ± SD. Different lowercase letters indicate significant difference (*P* < 0.05).

PGI, MDH, G-6-PDH and 6-PGDH exist in EMP, TCAC and PPP, respectively. PGI activities (Figure 2(A)) declined (*P* < 0.01) to 48% and 21% of the control at 10% and 30% water loss, respectively. However, further increase of water loss caused significant increase (*P* < 0.01) in PGI activities with 60% and 76% of the control at 50% and 70% water loss, respectively. It was clearly seen that MDH activities (Figure 2(B)) showed a drastic decline (*P* < 0.01) as the water loss increased. At 30% water loss, the activity dropped to only 2% of the control, and no enzyme activity was detected at 50% and 70% of water loss. The activities of G-6-PDH and 6-PGDH (Figure 2(C)) remained unchanged 10% of water loss as compared to the control. However, with the further increase of water loss in the fruit bodies, the activities increased gradually (*P* < 0.01). The activities of G-6-PDH and 6-PGDH at 30%, 50% and 70% water loss were 1.50-, 2.07- and 3.80-fold higher than those in the control, respectively.

Aerobic respiration is mainly carried out via EMP-TCAC. Nulton-Persson and Szweda [21] and Tretter and Adam-Vizi [22] point out that those enzymes in TCAC are sensitive to H_2_O_2_ and oxidative stress. In our study, the performance of MDH activities in conditions of water loss indicated that MDH was susceptible to drought stress, which is in line with these earlier findings. Our results also suggested that TCAC decelerated and further ceased its function with the increase in the intensity and duration of the drought stress. Decelerated TCAC could cause the accumulation of pyruvate, which could have restrained the EMP through feedback inhibition and could have led the PGI activities to decrease at 0%–30% water loss. On the other hand, the increase in PGI activity between 50% and 70% water loss may be related to polysaccharide biosynthesis.[23,24]

PPP can operate either aerobically or anaerobically and is of great importance to biosynthesis, as well as catabolism. Moreover, it has a protective effect against ROS and stress. Juhnke et al. [25] confirmed that mutants of yeast lacking PPP were sensitive to H_2_O_2_ and oxidative stress. In the present study, the enhanced activities of G-6-PDH and 6-PGDH meant that PPP offered more protection in conditions of drought stress. This could be considered to be of particular importance for the survival of the fruit bodies of *A*. *auricula* during drought stress.

Under drought stress, an organism must be able to protect itself against excess ROS to survive. The elimination of ROS and the reduction of oxidized intermediates resulting from membrane lipid peroxidation depend directly or indirectly on the reducing power,[18,25–27] as NADH, NADPH, FADH_2_, etc., which are electron donors and can take part in the scavenging of ROS. The reducing power of NADH and NADPH, superoxide dismutase (SOD) and peroxidase (POD), can remove O_2_·^−^ and H_2_O_2_, respectively.[28] Furthermore, the reducing power of these compounds and enzymes can regenerate other antioxidants, e.g. ascorbic acid (AsA) and reduced glutathione (GSH).[29] The major part of the compounds with reducing power are generated in the EMP-TCAC and PPP. In our study, despite the fact that EMP-TCAC brought about deficiency of NADH, PPP increased the supply of NADPH. Our previous study [30] revealed that the activities, or the level of some antioxidants (SOD, catalase, POD, AsA and GSH) in fruit bodies of *A*. *auricula-judae*, increased under drought stress. Therefore, the cooperation between NADPH and antioxidants would be more efficient in the scavenging of excess ROS induced by drought stress.

Based on the changes of soluble carbohydrates and respiratory enzymes, we could conclude that PPP played a principal role in protecting the fruit bodies of *A*. *auricula-judae* against drought stress.

## Conclusions

The results from this study showed that under drought stress, EMP-TCAC decreased sharply to zero, but PPP was significantly enhanced in the fruit body of *A. auricula*. PPP could produce sufficient reducing power to remove ROS, and hence enable the fruit body to survive the drought stress. Moreover, soluble polysaccharides may be helpful to protect the fruit body in further drought stress.
